# Modulation of gut microbiota mediates berberine‐induced expansion of immuno‐suppressive cells to against alcoholic liver disease

**DOI:** 10.1002/ctm2.112

**Published:** 2020-08-13

**Authors:** Sha Li, Ning Wang, Hor‐Yue Tan, Fan Chueng, Zhang‐Jin Zhang, Man‐Fung Yuen, Yibin Feng

**Affiliations:** ^1^ School of Chinese Medicine Li Ka Shing Faculty of Medicine The University of Hong Kong Pokfulam Hong Kong S.A.R P. R. China; ^2^ Division of Gastroenterology and Hepatology Queen Mary Hospital Department of Medicine Li Ka Shing Faculty of Medicine The University of Hong Kong Pokfulam Hong Kong S.A.R P. R. China

**Keywords:** alcoholic liver injury, berberine, granulocytic‐myeloid‐derived suppressor cell‐like population, gut microbiota

## Abstract

**Background:**

Berberine is an isoquinoline alkaloid compound derived from many herbs, which has been used extensively to improve liver function. But action mechanism of its hepatoprotection in alcoholic liver disease (ALD) is far from being clear.

**Aim:**

To investigate the underlying mechanism of berberine's therapeutic effect on ALD associated with gut microbiota‐immune system axis.

**Method:**

An animal model fed with ethanol that mimics drinking pattern ideally in ALD patients was established. Liver function was evaluated by biochemical test and histological examination. Immune cells were detected by flow cytometry and feces samples were collected for 16S rRNA gene amplicon sequencing.

**Results:**

We first reported the promising beneficial effect of berberine on ameliorating acute‐on‐chronic alcoholic hepatic damage and explored the underlying mechanism involving gut microbiota‐immune system axis. Notably, berberine activated a population with immune suppressive function, defined as granulocytic‐ myeloid‐derived suppressor cell (G‐MDSC)‐like population, in the liver of mice with alleviating alcohol‐induced hepatic injury. Berberine remarkably enhanced the increase of G‐MDSC‐like cells in blood and liver and decreased cytotoxic T cells correspondingly. Suppression of G‐MDSC‐like population significantly attenuated the protective effect of berberine against alcohol. Berberine activated IL6/STAT3 signaling in in vitro culture of G‐MSDCs‐like population, while inhibition of STAT3 activity attenuated the activation of this population by berberine. Moreover, berberine changed the overall gut microbial community, primarily increased the abundance of *Akkermansia muciniphila*. Of note, depletion of gut microbiota abolished the inducing effect of berberine on G‐MDSC‐like population, and attenuated its hepatoprotective effect against alcohol in mice, suggesting intestinal flora might be involved in mediating the expansion of this protective population.

**Conclusion:**

Collectively, this study delivered insight into the role of immunosuppressive response in ALD, and facilitated the understanding of the pharmacological effects and action mechanisms of berberine.

## Graphical abstract

- Berberine showed promising therapeutic effect on acute‐on‐chronic alcoholic liver injury.
- Gut microbiota‐immune system axis is involved in the mechanism underlying berberine's hepatoprotection.
- Berberine could maintain the expansion and function of G‐MDSC‐like cells by activating IL‐6/STAT3 pathways and mediating gut microbiota.

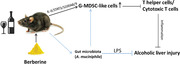

## 1 INTRODUCTION

Alcoholic liver disease (ALD) is considered to be a major factor of liver cirrhosis and liver‐related death all around world.^1^ Currently, treatment of ALD principally relies on abstinence, and no universally accepted therapy can inhibit lesion progression toward cirrhosis in active heavy drinkers.^2^ Accumulative evidence has indicated the significant role of immune system in the pathological process of ALD.^3^ Both innate and adaptive immune responses were activated in response to alcohol exposure, as evidenced by the infiltration of macrophages, neutrophils, B cells, and T cells, and resulting in hepatic inflammation via the induction of pro‐inflammatory cytokines.^3^, ^4^ Suppressing the infiltration of inflammatory cells could decrease the liver inflammation and thus impede the progression of ALD.

Myeloid‐derived suppressor cell (MDSC) consisted of a variety of immature myeloid cells, is primary component of the immune‐suppressive network.^5^ It was artificially classified as granulocytic‐MDSC (G‐MDSC) and monocytic‐MDSC (M‐MDSC) in rodents according to the relative expression level of Ly6G and Ly6C.^5^ G‐MDSC (CD11b^+^Ly6G^+^) and M‐MDSC (CD11b^+^Ly6G^−^Ly6C^high^) show different morphology, and exert immunosuppressive capacities via various pathways.^6^ Both M‐MDSC and G‐MDSC were believed to facilitate the escape of cancer cells from immune surveillance via T cells suppression decades ago, but until recently, their functional importance in the immune system has been more appreciated.^7^

MDSC expands and accumulates under most inflammatory circumstances, generating a variety of pro‐ and anti‐inflammatory cytokines in different settings.^8^, ^9^, ^10^ The role of M‐MDSC and G‐MDSC subsets in non‐cancer related diseases is determined by specific inflammation and timing.^11^ Of note, at present, the classification of CD11b^+^Ly6G^+^ population was still debatable because both G‐MDSC and neutrophils have CD11b and Ly6G expression on cell surface.^12^ Due to the similarity in morphological, phenotypic, and functional, confusion in investigating their roles in inflammatory diseases are generated.^13^ Generally, CD11b^+^Ly6G^+^ cells with T cells inhibition activity are regarded as G‐MDSC. Some types of neutrophils having immune‐suppressive effects might also be included.^13^ G‐MDSC might signify new phenotypes of neutrophils with immunosuppressive function.^14^ Thus, this neutrophil‐like cells, having the expression of CD11b^+^Ly6G^high^Ly6C^int^ with T cells inhibiting effects, are proposed to be labeled as G‐MDSC‐like cells. Our recent study has demonstrated this population showed a protective effect in responding to acute alcohol consumption.^15^ However, the role of this G‐MDSC‐like cells in the chronic ALD remains unclear.

Berberine, an alkaloid derived from the *Coptis chinensis* species with a long history of use in Chinese medicine, has been used widely as a hepatoprotective agent by many studies, most of which had focused on liver cancer, fatty liver disease, and hepatic fibrosis induced by toxins such as CCl_4_.^16^, ^17^, ^18^, ^19^, ^20^ It was also documented in possessing therapeutic efficacy on liver damage induced by alcohol abuse in animal studies.^21^ However, the therapeutic effect of berberine on hepatic damage induced by acute‐on‐chronic alcohol feeding is unknown. Model with acute‐on‐chronic alcohol feeding well mimics drinking pattern in ALD patients. Since drinking pattern significantly affects alcoholic liver damage, appropriate animal models are crucial to uncover the underlying mechanism and test new therapeutic agents.^22^

In present study, hence, a mice model with acute‐on‐chronic alcohol consumption created by Bertola et al. was adopted.^22^ Given the critical role of G‐MDSC‐like population in against inflammation, we inferred that berberine may mediate ALD associated with G‐MDSC‐like population. The participation as well as profile of G‐MDSC‐like cells and related immune cells were systemically investigated. Increasing evidence showed that intestinal flora composition was changed after alcohol administration, further influencing gut permeability and leading to injury.^23^, ^24^, ^25^ Modulating intestinal microbiota composition via pharmacological agents might be the promising direction for treatment of ALD.^26^ As berberine is treated as a potent regulator on intestinal flora,^27^, ^28^, ^29^ the role of intestinal bacteria changes in regulating G‐MDSC‐like cells was also examined.

## 2 MATERIALS AND METHODS

### 2.1 Reagents and antibodies

Murine IL‐6 cytokine was purchased from R&D Systems. Murine GM‐CSF cytokine GM‐CSF was purchased from Abcam. Anti‐Ly6G (1A8) was purchased from eBioscience. STAT3 inhibitor was purchased from BioVision. Ethanol was from Sigma–Aldrich. Anti‐phosphorylated STAT3 (p‐STAT3) was purchased from CST. APC‐CD11b, FITC‐Ly6G, and PE‐Cy7‐Ly6C antibodies were purchased from BioLegend. APC‐CD3, FITC‐CD4, and APC‐Cy7‐CD8 antibodies were purchased from BD Biosciences. Berberine hydrochloride was purchased from Sigma–Aldrich. Lieber‐DeCarli liquid diet was purchased from Bio‐Serv.

### 2.2 Animal treatment

C57BL/6J male mice (6‐8‐week‐old) were used in present study. Animal model was established according to the methods described in literature^22^ with minor modifications. All experimental mice had control liquid diet (item no. F1258SP, Bio‐Serv) ad libitum first. Then, after 5 days, mice are divided into ethanol‐fed and control groups. Ethanol liquid diet (item no. F1259SP, Bio‐Serv) containing 5% (v/v) ethanol was treated to ethanol‐fed groups, while control groups received the isocaloric control liquid diet (item no. F1258SP, Bio‐Serv). To have a longer observation, the period of this model was extended to 33 days with several binges. Ethanol‐fed mice were treated ethanol solution (5 g/kg body weight) via gavage at days 11, 22, and 33 while pair‐fed control mice received isocaloric maltose dextrin solution. Those ethanol‐fed mice were further divided into several groups, including ethanol model group, and berberine groups with different doses. Berberine groups were treated with berberine (10, 50, 100 mg/kg) via gavage every day, while ethanol model group received isovolumetric vehicle. Afterward, some of the mice of different groups were sacrificed to collect samples after binge treatment. The scheme of animal treatment was drawn in Figure S1. All procedures involving animals in this study were approved by the CULATR of The University of Hong Kong, Hong Kong.

### 2.3 Preparation of the cell suspension

Cells were isolated and prepared, as described previously.^30^, ^31^ (a) After the mice had been anesthetized with the mixture of ketamine and xylazine, the blood samples were collected via cardiac puncture, and then were mixed with heparin quickly. (b) Liver tissue was cut into small pieces and collected with RPMI 1640 containing collagenase solution. Afterward, collected solution was digested with moderate shaking for 0.5 h at 37°C. Then cell pellets were further re‐suspended in culture medium. Afterward, percoll density gradient centrifugation was used to separate mononuclear cells. (c) Spleen tissue was homogenized and subsequently collected with RPMI 1640. (d) Bone marrow cells were obtained from tibias as well as femurs of the mice. Ficoll density gradient centrifugation was applied to separate mononuclear cells from bone marrow. All red blood cells in cell population collected from liver tissue, spleen tissue, and blood were deleted by treating with lysis buffer and then washed with PBS for antibodies staining. The detailed protocol of this section is shown in Figure S2.

### 2.4 Flow cytometry

Obtained single‐cell suspension was stained with CD11b APC, Ly6G FITC, and Ly6C PE‐Cy7 or CD3 APC, CD4 FITC, and CD8 APC‐Cy7 antibody to detect MDSCs and T cells for 15 min in the dark. Corresponding isotype antibodies were used as controls. Afterward, re‐suspended cells in phosphate buffer solution (PBS) containing 3% FBS (Sigma–Aldrich) subjected to flow cytometry. The cellular population was analyzed on Canto flow cytometer.^32^ FlowJo software was applied for further data processing.

### 2.5 Remove in vivo MDSC‐like cells

Isotype IgG2b antibody or Anti‐Ly6G (1A8, 120 µg per mouse) was injected into mice intraperitoneally every 3 days throughout the whole experimental period.

### 2.6 In vitro cell culture

MDSC‐like population was sorted from bone marrow cells of mice by Aria I flow cytometer^32^ and afterward cultured in RPMI 1640 medium. In some in vitro culture, cytokine factors such as GM‐CSF (80 ng/mL), IL‐6 (40 ng/mL), or STAT3 inhibitor (50 µM) were accordingly supplemented into the culture medium. For in vitro experiment, berberine (10 µM) was co‐cultured for 24 or 48 h.

### 2.7 Cytokines contents measurement

LEGENDplex™ Mouse Inflammation Panel (San Diego, USA, 13‐plex) was used to measure the level of cytokines in the serum. The measurement was performed on Flow Cytometer according to the instructions provided within the product.

### 2.8 Serum aminotransferase activity measurement

The activities of aminotransferase including alanine aminotransferase (ALT) and aspartate aminotransferase (AST) in the serum were examined by using kit (Biovision US) instructed by manufacturer's guidelines.

### 2.9 Liver histology

The liver tissues were fixed in 10% neutral‐buffered formalin and then cut paraffin sections at a thickness of 5 µm by a Leica RM 2016 rotary microtome (Shanghai, China). Afterward, stained the processed tissue with hematoxylin and eosin. Then histological images were visualized using Microscopes (Olympus BX43). The scoring of liver injury was measured by criteria according to our previous paper.^33^

### 2.10 Quantitative real‐time PCR

All procedures were done accordingly to Wang et al.^34^ Total RNA was extracted with TRIzol reagent (Invitrogen). Afterward, PrimeScript Real‐time Reagent kit was used to synthesize cDNA (TaKaRa). Real‐time PCR was performed using SYBR Green Master Mix (TaKaRa) and quantified by PCR system (Roche Light Cycler 480, USA). And β‐actin was selected as housekeeping genes control. All of the primer sequences used are shown in Table S1.

### 2.11 Gut flora identification

Fecal samples of mice were collected in autoclaved tubes and stored at −80°C before further processing. DNA of fecal bacteria was extracted using the QIAamp DNA Stool Mini Kit following instructions (Qiagen, Cat# 51504). The DNA concentration were detected by 7415 Nano spectrophotometer, and the 260/280 and 260/230 measurement ratio as well as DNA gel electrophoresis were detected to check and quantify the purity of the DNA sample. Then, qualified DNA samples were obtained and stored at −20°C before use. The amplicon library was constructed via the amplification of V3–V4 hypervariable regions of 16S rRNA gene sequences using the primers (319F/806R). PCR amplification program was performed with DNA polymerase in a thermocycler under the following steps: initial denaturation at 98°C for 2 min, followed by 30 cycles of 98°C for 15 s, 58°C for 15 s, 72°C for 15 s, and a final extension of 72°C for 3 min. The qualities of PCR products were assessed by an Agilent bioanalyzer 2100 system (Agilent, CA,USA), and the amplicon pool of PCR products was prepared with Ampure XP beads, subsequently the pooled PCR products were sequenced at Beijing Genomics Institute. Co., Ltd., with the Illumina MiSeq sequencing system.

After basic cleaning and quality filtering in QIIME (http://qiime.org/), high‐quality clean tags were obtained. Sets of sequences with 97% identity as a threshold were defined as an Operational Taxonomic Unit (OTU) by using software USEARCH (v7.0.1090). OTU representative sequences were performed by RDP Naïve Bayesian Classifier v.2.2. The data analysis and visualization of different microbiota communities including OTU number (Venn diagram), OTU composition comparisons (PCA analysis), and the community compositions at various classification of Phylum, Class, Order, Family, Genus, and Species were performed with R (v3.1.1) software. Statistical significance of microbial communities between the taxonomic groups of samples was calculated by using Metastats and R (v3.1.1).

### 2.12 Cecal content transplantation

Mice from berberine groups, having ethanol liquid diet for 10 days plus once alcohol binge, were euthanized and sacrificed. Afterward, the ceca were moved out in sterile conditions and harvested in 80% glycerol/PBS solution. After centrifugation at 800 rpm for 2 min, supernatants were collected and stored at −80°C. Afterward, we treated diluted cecum solution (0.2 mL) per mouse every day.

### 2.13 Generation of pseudo germ‐free mice

Pseudo germ‐free (PGF) mice were obtained following protocol of previous literature.^28^ Mice were administrated with Terramycin (300 mg/kg), erythromycin (300 mg/kg), and cefadroxil (100 mg/kg) twice a day for three consecutive days. To check germ‐free status of mice, bacteria cultures of feces were performed on Luria‐Bertani agar. Control feces were obtained from mice without antibiotic treatment.

### 2.14 Bacteria cultures

All procedures were conducted aseptically with sterilized tools and solutions. Fifty micrograms of feces from mice were weighted. Based on optimization by a range of dilutions, those collected fecal samples were re‐suspended in sterile normal saline solution and diluted for 10 times. After dilution, 100 uL of fecal suspension were evenly spread on sterile plates of Luria‐Bertani agar culture medium. Then inoculated plates were incubated at 37°C and single colonies numbers were calculated at 24 and 72 h.

### 2.15 Western blot

All procedures of western blot were conducted according to the methodology described in the literature.^34^ Briefly, RIPA lysis buffer with phosphatase inhibitor and cocktail proteinase inhibitor was used to extract cellular protein. SDS‐PAGE gel was used to separate protein lysates and then transferred to PVDF membranes. After blocking to prevent nonspecifically binding, the suitable cut membranes were incubated with appropriate dilutions of primary antibody overnight followed by secondary antibodies with HRP‐labeled. Then, blots were analyzed with chemiluminescence detection.

### 2.16 Statistical analysis

We presented the data as mean ± SD. Comparisons among different groups were achieved by unpaired two‐way ANOVA or Student's *t*‐test. A *P*‐value of < .05 was considered statistically significant.

## 3 RESULTS

### 3.1 Berberine alleviated ALD in mice with acute‐on‐chronic alcohol feeding

**FIGURE 1:**
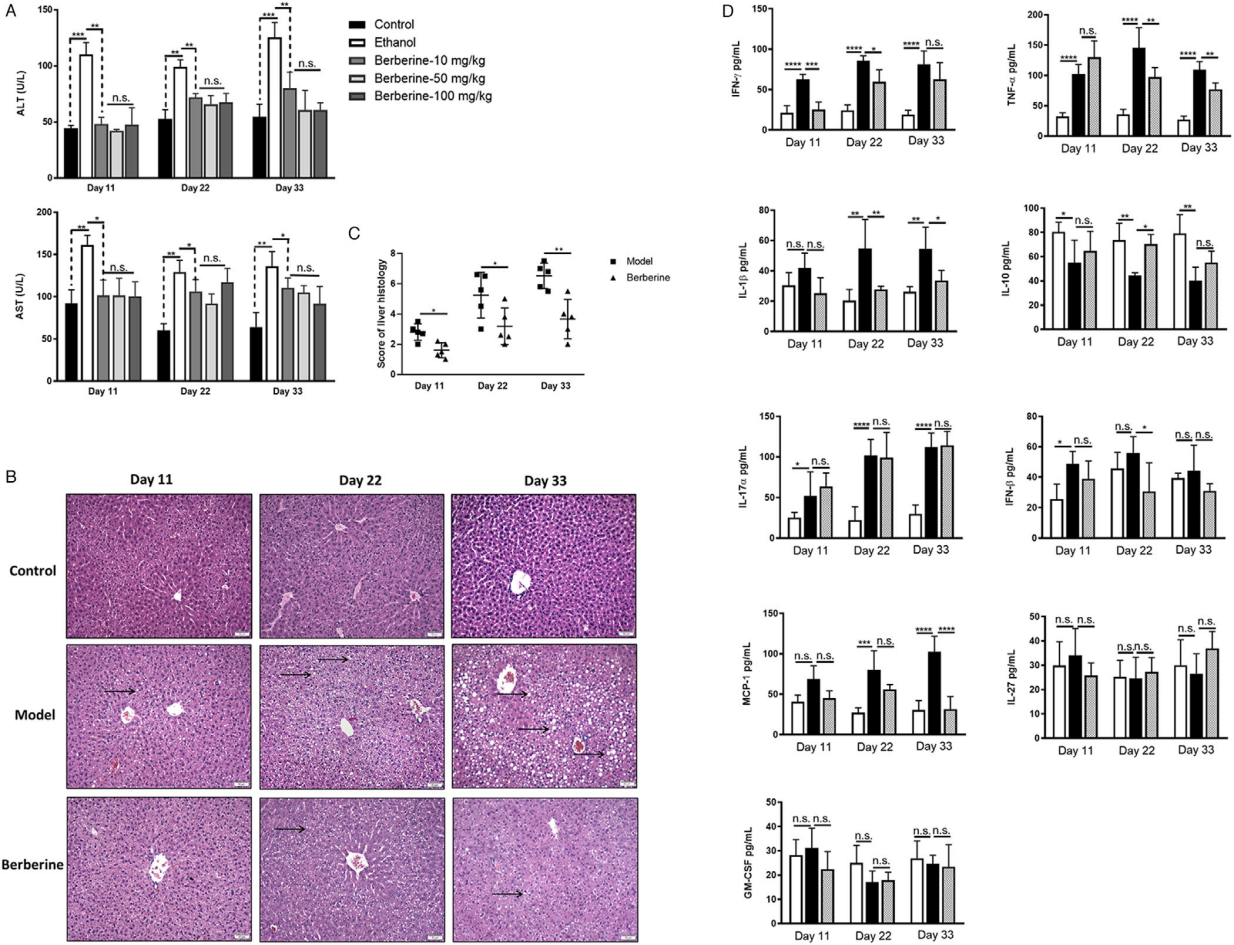
Berberine treatment significantly reduced alcoholic liver injury. Mice were divided into five groups, control group (N = 15) receiving control Lieber‐DeCarli diet and vehicle treatment, model group (N = 15) receiving ethanol Lieber‐DeCarli diet and vehicle treatment, low‐dose berberine group (N = 15, 10 mg/kg), medium‐dose berberine group (N = 15, 50 mg/kg), high‐dose berberine group (N = 15, 100 mg/kg) receiving ethanol Lieber‐DeCarli diet. Five mice of each group were sacrificed at day 11, day 22, and day 33, respectively. A, Serum ALT and AST level of mice from groups mentioned above at different time points. Berberine treatment significantly reduced the elevated serum ALT and AST levels at checked time points. No statistical differences among different dose groups of berberine were found. Thus, the dose of 10 mg/kg was used in the following experiments (B). Representative H&E staining images of livers from mice of control group, model group, and berberine group (10 mg/kg). The lipid deposition and steatosis are indicated by arrows. C, Scoring of liver histology. D, Serum contents of inflammatory cytokines including IFN‐γ, TNF‐α, IL‐1β, MCP‐1, IFN‐β, IL‐17A, IL‐10, IL‐27, and GM‐CSF determined by using LEGENDplex™ Mouse Inflammation Panel (13‐plex) with flow cytometer in mice from control group, model group, and berberine group. Berberine reduced the alcohol‐induced increased levels of IFN‐γ, TNF‐α, and IL‐1β. ^*^ *P* < .05, ^**^ *P* < .01, ^***^ *P* < .001, ^****^ *P* < .0001; n.s., not significant

Since mouse model of chronic ethanol feeding with binge (also named as NIAAA model) well mimics drinking pattern of humans, it has been widely used in studies of ALD.^22^ In this study, we established the NIAAA model with minor modification by prolonging the ethanol feeding time up to 33 days plus several acute alcohol gavages. The results indicated that berberine remarkably decreased the levels of ALT and AST in serum of mice treating for 11, 22, or 33 days, compared to mice from model group (Figure 1A). In our study, however, no statistical differences among berberine treatment groups of different doses (10, 50, and 100 mg/kg) were found (Figure 1A), suggesting that at the range of 10‐100 mg/kg, there is no evident dose‐response relationship. Besides, the histological H&E staining images further showed that berberine treatment significantly alleviated inflammation and hepatic steatosis (Figure 1B). Specifically, berberine treatment completely inhibited macrosteatosis and only slight microvesicular steatosis present in the liver of mice treated for 33 days (Figures 1B,C). As inflammatory cytokines and chemokines are crucial mediators of immuno‐response, we conducted simultaneous measurement of 13 cytokines/chemokines by using fluorescence‐encoded beads on flow cytometer. Serum inflammatory cytokines including TNF‐α, GM‐CSF, IFN‐β, IFN‐γ, MCP‐1, IL‐1β, IL‐1α, IL‐10, IL‐6, IL‐17A, IL‐12p70, IL‐27, and IL‐23 were examined. The results demonstrated that berberine treatment decreased significantly the contents of IFN‐γ, TNF‐α, and IL‐1β, while contents of other cytokines such as IFN‐β, MCP‐1, IL‐17A, IL‐27, IL‐10, and GM‐CSF were not influenced obviously (Figure 1D, data of IL‐23 and IL‐12p70 are not present as they were less than 2.5 pg/mL). Obtained data demonstrated the beneficial effect of berberine on resisting hepatic injury induced by long‐standing alcohol exposure.

### 3.2 Berberine induced G‐MDSC‐like cells expansion in NIAAA mice

**FIGURE 2:**
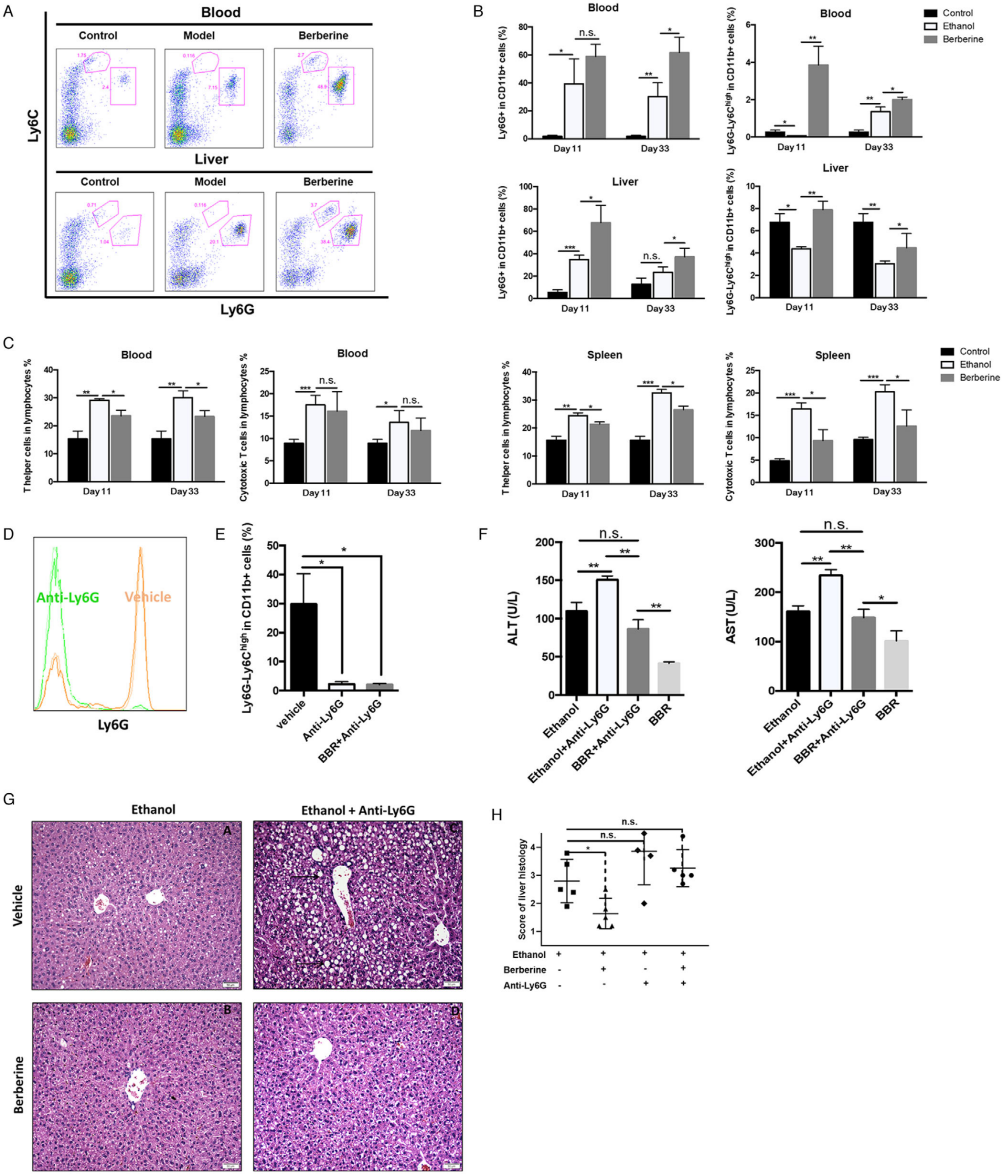
Berberine mediated the increase of G‐MDSC‐like cells to protect liver from alcohol‐induced injury. The populations of G‐MDSC‐like cells, M‐MDSC, or T cells in blood, liver, and spleen of mice were determined by flow cytometer. Berberine significantly promoted the increase of G‐MDSC‐like cells in liver and blood. A, The representative histogram images and quantification (b) of flow cytometric analyses of G‐MDSC‐like cells and M‐MDSC in blood and liver of mice of control group, ethanol model group, and berberine group. C, Quantification of flow cytometric analyses of T helper and cytotoxic T cells in blood and spleen of mice. Anti‐ly6G antibody or vehicle was treated to ethanol‐fed mice (N = 5 for each group). D, Representative histogram images and quantification (E) of flow cytometric analyses of G‐MDSC‐like cells in liver of mice treated with anti‐Ly6G or vehicle. F, Serum ALT and AST level of mice treated with anti‐Ly6G or vehicle. G, Representative H&E staining images of liver of mice treated with anti‐Ly6G or vehicle and scoring of histological damage (H). The lipid deposition and steatosis are indicated by arrows. ^*^ *P* < .05, ^**^ *P* < .01, ^***^ *P* < .001; n.s., not significant; BBR, berberine

Chronic hepatic injury caused by alcohol abuse was found mediated by the immune response in which both pro‐inflammatory and anti‐inflammatory leukocyte lineages were involved.^35^, ^36^ We first established the profile of the associated immune cells in mice with alcohol exposure (Figure S3). The immunosuppressive function of gated CD11b^+^Ly6G^high^Ly6C^int^ cells was demonstrated in our previous paper,^15^ which could support the definition of this population as G‐MDSC‐like cells. The proportion of related immune cells including G‐MDSC‐like cells, M‐MDSC, and T cells in spleen, liver, bone marrow, and blood of mice were examined by Canto II cytometry at selected point‐in‐time (5th, 11th, 17th, 22th, 28th, 33rd day). Results showed the population of M‐MDSC increased in liver and blood, while CD4/CD8^+^ T cells showed insignificant trend of changes (Figure S3). Strikingly, it indicated that amount of G‐MDSC‐like population always increased considerably after binge administration, while the amplitude was reducing (Figure 2A,B). Meanwhile, we found that berberine stimulated the increase of G‐MDSC‐like population in blood and liver significantly, especially at 33rd day (Figure 2A,B). Correspondingly, levels of CD4^+^/CD8^+^ T cells in spleen, blood, and liver in the mice from berberine group were lower than that of model group (Figure 2C). To explore if this expansion of G‐MDSC‐like population is participated in resisting alcoholic hepatic injury, anti‐Ly6G antibody was used. Injection of anti‐Ly6G antibody effectively removed hepatic CD11b^+^Ly6G^high^Ly6C^int^ cells (Figure 2D,E). Afterward, liver function was evaluated to assess if hepatic injury was aggravated when G‐MDSC‐like cells are absent. Serum levels of ALT and AST from mice receiving anti‐Ly6G antibody administration were significantly increased than that of vehicle group (Figure 2F). Meanwhile, as shown in histological images, much serious liver steatosis was observed in mice receiving anti‐Ly6G antibody (Figure 2G,H). Interestingly, we found that the protective effect of berberine was abrogated partially by G‐MDSC‐like population depletion, as demonstrated by the raised serum levels of aminotransferase as well as improved histological images (Figure 2F–H). It indicated that the mechanism underlying Berberine's positive performance involved this immunosuppressive population. We inferred that the increase of G‐MDSC‐like population possibly serve as a kind of self‐protection in response to alcohol exposure, but it would turn weak progressively if excessive alcohol intake is prolonged, which thus resulted in persistent inflammation and tissue damage. The hepatoprotection of G‐MDSC‐like population responding to alcohol intake would be suppressed in chronic context, while berberine treatment facilitated this expansion to resist severe ALD.

### 3.3 Mechanism underlying berberine‐mediated G‐MDSC‐like cells expansion

**FIGURE 3:**
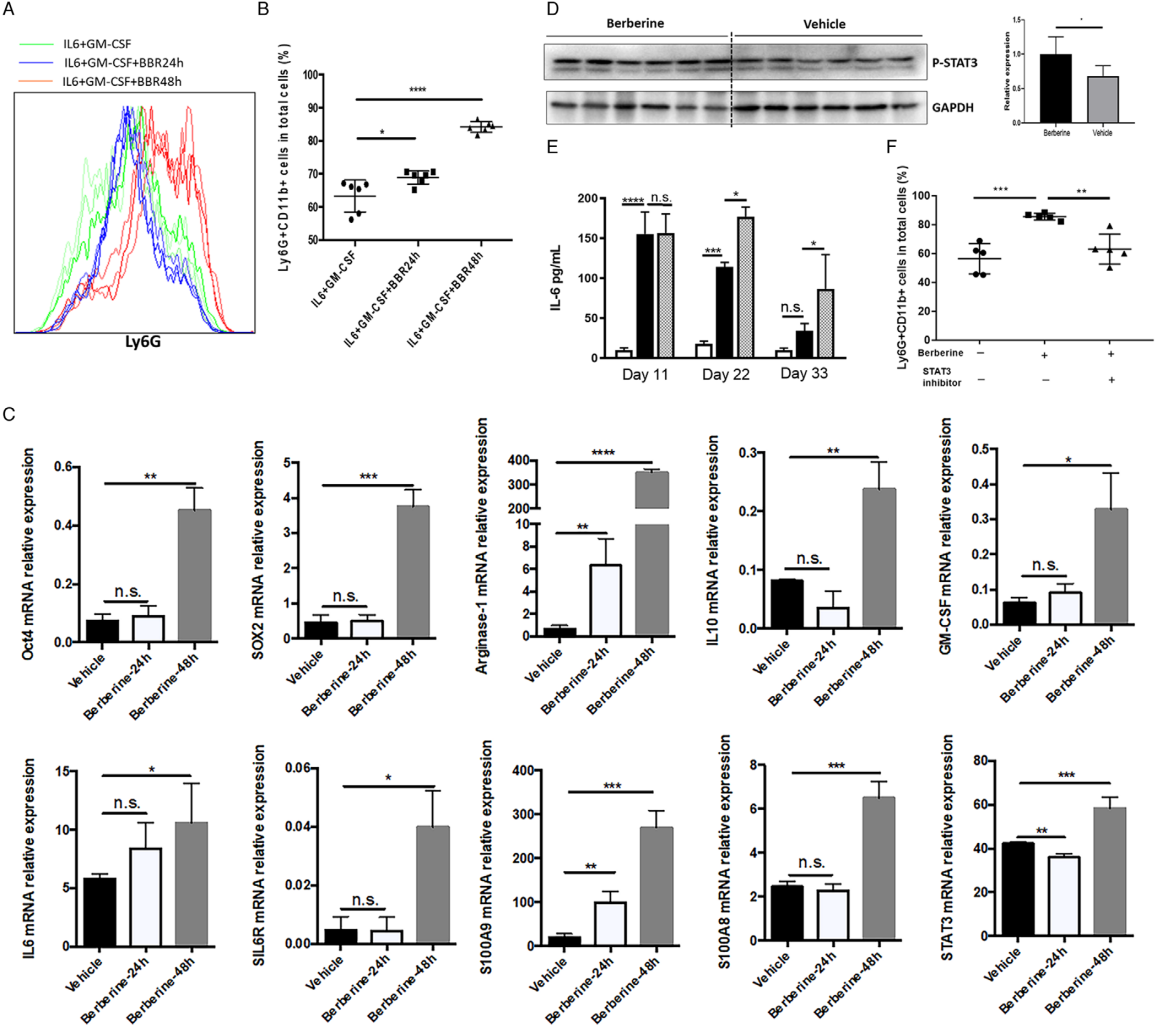
Berberine sensitized G‐MDSC‐like population by activating IL‐6/STAT3 pathways. Bone marrow derived cells were co‐cultured with berberine (10 µM) for 24 or 48 h in RPMI1640 supplemented with 10% fetal bovine serum, IL‐10, and GM‐CSF. Afterward, cells were collected for population examination, total RNA and protein extraction. A, The representative histogram images, and quantification (B) of flow cytometric analyses of G‐MDSC‐like cells after 24 or 48 h in vitro culture. C, The m‐RNA relative expression level of targets including Oct4, SOX2, Arginase‐1, IL‐10, GM‐CSF, IL‐6, soluable IL‐6 receptor (SIL6R), S100A9, S100A8, and STAT3 in cells cultured in vitro. D, The protein expression level of p‐STAT3 determined by western blotting in cells cultured in vitro. E, The content of IL‐6 in serum of mice. F, The population of G‐MDSC‐like cells in the presence of STAT3 activation inhibitor. ^*^ *P* < .05, ^**^ *P* < .01, ^***^ *P* < .001, ^****^ *P* < .0001; n.s., not significant

To understand the mechanism of regulation on G‐MDSC‐like population mediated by berberine, myeloid cells containing G‐MDSC‐like cells and their progenitors or precursors from the bone marrow of naïve mice have been isolated and then co‐cultured with berberine in vitro. In the culture, to assist the survival and differentiation of those isolated myeloid lineage, two cytokines GM‐CSF and IL‐6 were supplemented. After co‐cultured with berberine or vehicle for 24 or 48 h, CD11b^+^Ly6G^+^ population was determined by flow cytometry. It indicated that berberine could significantly accelerate the differentiation of G‐MDSC‐like population (Figure 3A,B). The mRNA level of Sox2 and Oct‐4, which are two vital reprogramming mediators for the differentiation of myeloid progenitors, was significantly increased by berberine with 48 h treatment (Figure 3C), indicating the preferential differentiation from myeloid precursors to G‐MDSC‐like populations induced by berberine. The expression levels of mRNA of genes relating to immune‐suppression capacity including IL‐10, arginase‐1, and GM‐CSF were remarkably improved by berberine treatment (Figure 3C). Furthermore, berberine significantly enhanced the expression of S100A8, S100A9, STAT3, IL‐6, and IL‐6R in these myeloid cells (Figure 3C). The expression level of p‐STAT3 in the cultured cells was also increased by berberine treatment for 48 h (Figure 3D). This suggested that IL‐6/STAT3 signaling pathway participated in berberine‐mediated increase of G‐MDSC‐like population.

IL‐6 interacts with IL‐6 receptor expressing on MDSC‐like cells and then activates the pathway to expand.^30^, ^37^ The serum content of IL‐6 was elevated in mice receiving alcohol feeding with reduced amplitude of increase along with treatment time, similar as the alternation of G‐MDSC‐like population as previously described (Figure 3E). Thus, we inferred that IL‐6 might serve as the driver of stimulating G‐MDSC‐like cells. It is wakened gradually when alcohol consumption was persisted. Meanwhile, as shown in Figure 3E, berberine administration significantly upregulated IL‐6 level. More importantly, the increase of G‐MDSC‐like cells by berberine was significantly abrogated in the presence of STAT3 activation inhibitor (Figure 3F). Hence, it was indicated that berberine promoted the differentiation into G‐MDSC‐like cells partly via IL‐6/STAT3 pathways.

### 3.4 Berberine mediated disordered gut microbiota induced by alcohol

**FIGURE 4:**
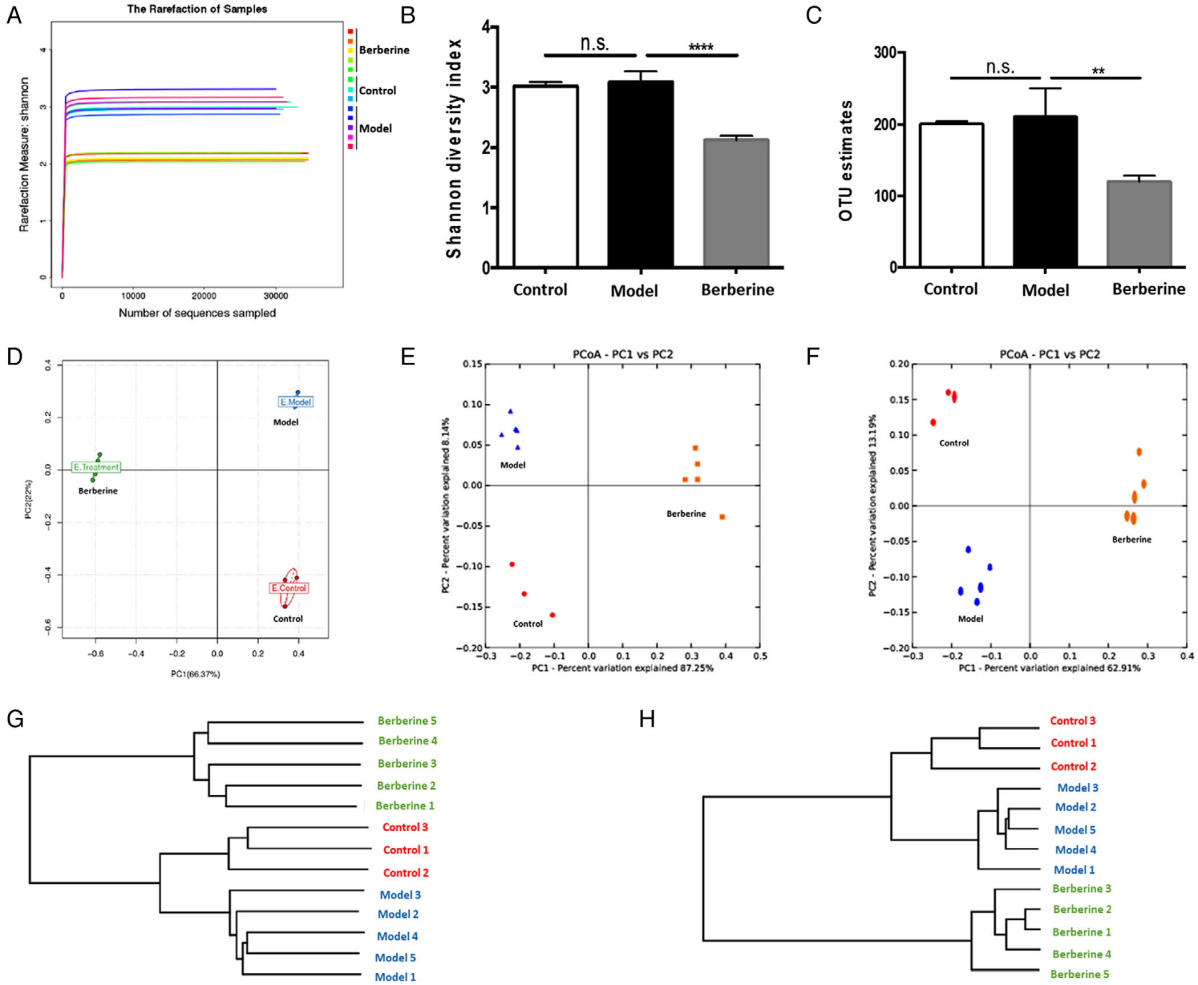
Diversity and richness of the gut microbiota in mice and responses of the structure of the gut microbiota to berberine treatment. Chromosomal DNA was extracted from feces sample of mice. After sample quality tests, 13 samples (N = 3 for control group, N = 5 for model group, N = 5 for berberine group) were qualified. Gene amplification and sequencing of the V4 region of 16S rRNA were then performed by The Beijing Genomics Institute. A, Shannon curve. B, Rarefaction OUT estimates. C, Shannon index. D, PCA score plot. E, PCoA score plot based on unweighted and weighted Unifrac metrics. F, Unweighted pair group method with arithmetic mean (UPGMA) based on unweighted Unifrac (F) or weighted Unifrac (G) analysis. ^**^ *P* < .01, ^***^ *P* < .001; n.s., not significant

Accumulative evidence has shown that gut microbiota is of great importance in the progression of a variety of liver diseases including ALD.^38^, ^39^, ^40^ Berberine is considered to have low bioavailability owing to its low absorption rate.^28^ The hepatoprotection of berberine was, thus, deliberated indirect. Therefore, berberine's effect in mediating diversity and composition of gut microbiota in ALD was expected. We extracted cDNA from fecal samples of mice. After testing sample quality, total 13 samples (3 from control mice, 5 from model mice, 5 from berberine‐treated mice) were qualified for further analysis. The data obtained in our preliminary experiment showed low noise, and standard deviation is limited. Thus, we adopted this sample size in gut microbiota characterization by power analysis (5% significance, two‐sided, 80% power, SN ration = 2.8). Then, gene amplification was performed and V4 region of 16S rRNA were sequenced. As shown in Figure 4A, the sequencing depth we had was adequate to have most intestinal microbes in samples. Insignificant differences were observed in diversity and richness of gut microbiota between the control group and model group, while berberine treatment reduced both richness and diversity significantly, as evidenced by Shannon diversity index and OTUs (Figure 4B,C). To analyze the differences of OTUs composition among different groups, Unifrac distance‐based Principal Coordinate Analysis (PCoA) and principal component analysis (PCA) were used to construct 2D graph to figure out factors primarily contributing to this difference. PCA scores indicated that, compared with the control group, the intestinal flora in model group presented a remarkable structural shift along the first principal component (PC1) (Figure 4D). In the meantime, berberine was observed to alter variations along PC1 significantly induced by alcohol (Figure 4D). Both weighted (Figure 4E) and unweighted (Figure 4F) Unifrac analysis indicated obvious alternations in the overall intestinal flora community after berberine treatment. Furthermore, UPGMA based on unweighted Unifrac (Figure 4G) or weighted Unifrac (Figure 4H) analysis, a kind of hierarchical clustering approach by means of average linkage to interpret the distance matrix generated by beta diversity, also displayed significant regulation of berberine on the intestinal flora.

**FIGURE 5:**
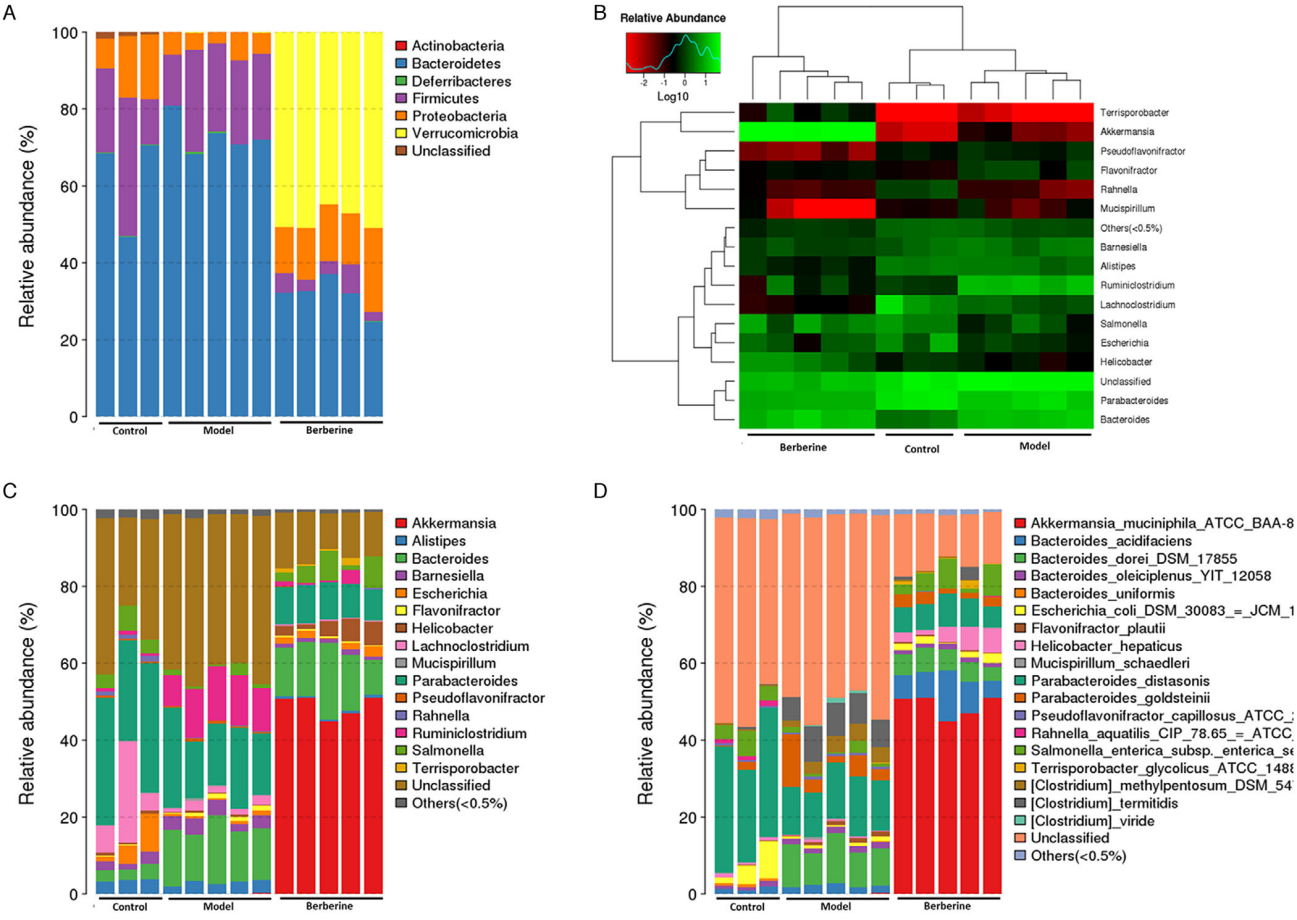
Berberine regulated alcohol‐induced disordered gut microbiota. A, The taxonomic composition distribution in samples of phylum‐level. B, Log‐scaled percentage heat map of genus‐level. C, The taxonomic composition distribution in samples of genus‐level. D, The taxonomic composition distribution in samples of species‐level

Then we aimed to identify specific gut flora that potentially mediate the hepatoprotection of berberine on ALD. At the phylum level, we observed the phylum of *Verrucomicrobia* abundance was higher than that of berberine group (Figure 5A). Currently, the only identified member of this phylum is mucus‐degrading bacterium *A. muciniphilais*.^41^ Additionally, the abundance of *Proteobacteria* was diminished by alcohol exposure, while berberine reverse it to normal level significantly (Figure 5A). Although this phylum occupies a minor proportion in normal gut flora, the association between an abnormal alteration of Proteobacteria and a compromised ability to sustain a balanced intestinal microbial community has been proposed, which deservers further investigation.^42^ At the genus level, as shown by the heat map, there are 15 key genus have different abundance in those groups (Figure 5B). Among them, Terrisporobacter and Helicobacter were increased, while Pseudoflavonifractor, Mucisirillum, Alistipes, Ruminiclostridium, and Lachnoclostridium were decreased by berberine treatment. Notably, *A. muciniphila*, serving a critical role in keeping integrity of gut barrier, is the most striking flora influenced by berberine administration (Figure 5C,D). As demonstrated by a previous study, alcohol exposure reduced *A. muciniphila* abundance in both murine model and ALD individuals, and *A. muciniphila* administration could alleviated hepatic damage induced by alcohol.^43^ To sum up, berberine might attenuate ALD via regulation of flora communities, particularly by enhancing *A. muciniphila* abundance.

### 3.5 Berberine‐regulating gut flora mediates the G‐MDSC‐like cells as well as liver protection in NIAAA models

**FIGURE 6:**
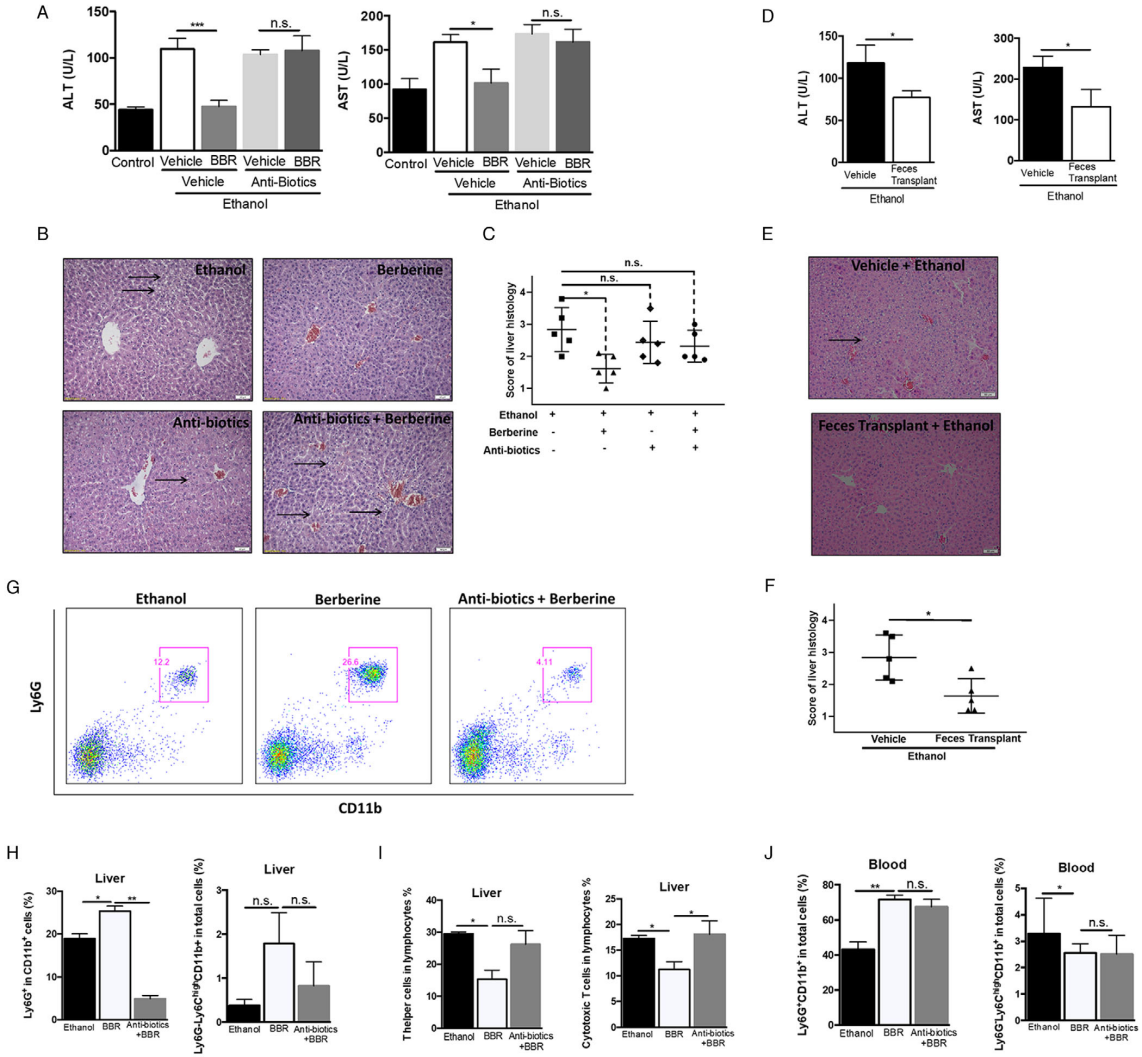
Gut microbiota is actively involved in the beneficial effect of berberine. PGF mice were built up by antibiotics treatment. These PGF mice received ethanol‐ Lieber‐DeCarli diet with berberine treatment (10 mg/kg) or vehicle treatment for 11 days (N = 5 for each group). Then liver injury was evaluated and the population of G‐MDSC‐like cells in mice were examined by flow cytometer. A, Serum ALT and AST level of mice from control group, ethanol model group, berberine group, berberine with antibiotics group, and vehicle with antibiotics group. B, Representative H&E staining images of livers and scoring of histological damage (C) from mice of ethanol model group, berberine group, berberine with antibiotics group, and vehicle with antibiotics group. Moreover, cecal contents were collected from mice of berberine group and then treated to mice receiving ethanol‐Lieber‐DeCarli diet for 11 days (N = 5 for each group). D, ALT and AST level of mice from ethanol model group and feces transplant group. E, Representative H&E staining images of liver and scoring of histological damage (F) from mice of model group and feces transplant group. G, The representative histogram images and quantification (H) of flow cytometric analyses of G‐MDSC‐like cells in liver of mice from ethanol group, berberine group, and berberine with antibiotics group. I, The population of T helper and cytotoxic T cells in liver of mice from ethanol group, berberine group, and berberine with antibiotics group. J, The population of G‐MDSC‐like cells and M‐MDSC in blood of mice from ethanol group, berberine group, berberine with antibiotics group. ^*^ *P* < .05, ^**^ *P* < .01; n.s., not significant; BBR, berberine

To further investigate whether berberine‐mediated intestinal flora is participated in G‐MDSC‐like population‐associated hepatoprotection, we produced PGF mice with berberine administration to observe if gut microbiota deletion would abolish its hepatoprotective effect. After treating mice with antibiotics for 3 days, feces of mice were collected to culture in vitro for checking the germ‐free status. As shown in Figure S4, mice treated with antibiotics present PGF performance. Figure 6A‐C demonstrated that in the condition of PGF, berberine did not show any positive effect on alcohol‐induced hepatic injury. In addition, we transplanted cecal content from mice treated with berberine to mice having alcohol liquid diet in order to explore if the hepatic injury could be diminished. Compared with vehicle group, significant lower serum levels of ALT and AST were observed in group receiving cecal content transplantation (Figure 6D). The microvesicular steatosis and hepatocyte injury were improved after transplanting cecal content (Figure 6E,F). Because berberine‐mediated expansion of G‐MDSC‐like cells was considered to facilitate its protective effect against alcohol, we further explore whether intestinal flora depletion would abrogate berberine's regulation on G‐MDSC‐like population. Interestingly, berberine failed to raise population of hepatic CD11b^+^Ly6G^+^ PGF mice (Figure 6G,H), and CD8^+^ T cells but not CD4^+^ T cells were significantly increased (Figure 6I). The population of CD11b^+^Ly6G^+^ between control mice and berberine‐treated PGF mice showed no significant difference in blood (Figure 6J). The population of CD11b^+^Ly6G^−^Ly6C^high^ was not altered obviously in both liver (Figure 6I) and blood (Figure 6J). We demonstrated that the positive performance of G‐MDSC‐like population in orchestrating the influence of berberine in ALD was regulated by gut flora.

## 4 DISCUSSION

Berberine has been demonstrated as hepatoprotective agent; however, its efficacy on acute‐on‐chronic alcoholic hepatic damage remains unknown. It is considered that model of acute‐on‐chronic alcohol consumption is similar to drinking pattern of ALD patients. The vast majority of alcoholic patients have a recent excessive drinking history and chronic drinking for many years. Therefore, NIAAA model with acute‐on‐chronic alcohol feeding was adopted in our study.^22^ Berberine presented remarkable beneficial effect on alleviating hepatic damage induced by alcohol feeding, signifying its potency from bench to bedside for ALD therapy.

Accumulating studies have intended to understand the underlying mechanisms regulating MDSCs accumulation in tumors,^8^, ^44^ but it is only until recent decade, its role in several pathological conditions has been highlighted.^8^, ^9^, ^10^ However, one of challenges ahead in the study of MDSCs in inflammation and overt‐immune related disease is that G‐MDSC and neutrophils show similar phenotypes and surface markers, making it difficult to distinguish them.^45^, ^46^ In the gated CD11b^+^Ly6G^+^Ly6C^int^ population, inevitably, some mature neutrophils might be included. According to a rigorous nomenclature approach, therefore, we named this population as G‐MDSC‐like cells. Although in our study this subset has not been fully identified, our work nevertheless facilitates better understanding of insight into immunosuppression in ALD and reinforce the notion that MDSCs‐like population might be a self‐protective attempt of the body to alleviate the injury induced by sustained inflammation. Based on current available limited evidence, a complicated role was proposed on the participation of immunosuppression network including G‐MDSC‐like cells in ALD. On the one hand, this tolerogenic response may limit immunity and reduce subsequently hepatic damage. However, on the other hand, immunosuppression may impede eradication of pathogens and favors to chronic infections.^47^ Thus, it is crucial to elucidate the role of G‐MDSC‐like population in chronic context. In this study, we found G‐MDSC‐like cells always dramatically raised after acute alcohol administration, but the amplitude was decreasing. More importantly, we observed liver damage was aggravated significantly when G‐MDSC‐like cells are absent, indicating their protective role. These results signified that this self‐protection response of G‐MDSC‐like cells against alcohol‐induced hepatic injury would be progressively closed when alcohol exposure is persisted. Regulation on this cell population might be a promising strategy for ALD therapy.

We further noted that the positive effects of berberine were partially abolished with anti‐Ly6G treatment, signifying that G‐MDSC‐like cells participated in the action mechanism of berberine. Mechanisms underlying berberine's hepatoprotection have been extensively studied but are still far from being clear. Its hepatoprotective effect is generally attributed to the anti‐inflammation, anti‐oxidative stress, against endoplasmic reticulum stress, and reducing lipid accumulation capacities.^16^ This immunosuppression perspective might be new breakthrough of understanding berberine's pharmacological effects. To further understand berberine‐mediated increase and functioning of G‐MDSC‐like cells, we mainly focused on IL‐6, considering it serves as a distinguished cytokine of MDSCs expansion.^48^, ^49^, ^50^ We observed that IL‐6 level showed similar variation trend as population of G‐MDSC‐like cells responding to chronically alcohol administration. IL‐6 might be the main driver facilitating the increase of G‐MDSC‐like cells to resist hepatic damage induced by alcohol. Berberine activated IL6/STAT3 signaling in in vitro culture of G‐MSDCs‐like population, while inhibition of STAT3 activity attenuated the activation of this population by berberine. Furthermore, berberine upregulated the mRNA expression level of Sox2 and Oct‐4 significantly, which are two essential transcription mediators to preserve an undifferentiated status of stem cells. Berberine facilitates the preservation of progenitor‐like capacity of leukocytes via upregulating Sox2 and Oct‐4, and thereby promoting the differentiation into G‐MDSC‐like population.

Overwhelming evidence has emphasized the crucial role of intestinal flora in ALD pathogenesis. Excessive alcohol drinking causes altered composition of intestinal flora and dysbiosis, which is considered as a motivating force for the development of ALD.^32^ Augmented translocation of microbial by‐products such as lipopolysaccharides (LPS) due to impaired gut epithelial tight junctions and increased permeability are involved in ALD pathological process.^26^ As the leading action site of berberine is the gut, the impact of berberine on regulating intestinal microbiota composition greatly contributes to its pharmacological effects.^27^ In this study, we found that berberine regulated alcohol‐induced dysbiosis. Berberine significantly decreased richness and diversity of the gut flora and changed the overall composition of flora community. Diversity and richness of microbiota are generally associated with a healthy gut. As shown in another study,^51^ berberine caused intestinal flora dysbiosis as evidenced by the reduced observed species number and Shannon index, which is consistent with our results. Berberine possessed antibiotic‐like effects. It could modulate the composition of gut flora to a certain degree of dysbiosis. Of note, among the altered flora, *A. muciniphila* was the one that most remarkably influenced by berberine administration. *A. muciniphila* is a Gram‐negative anaerobic commensal that has a key role in maintaining integrity of gut barrier in a healthy individual.^52^ As demonstrated by a previous study, *A. muciniphila* abundance is remarkably decreased in both mice and humans after ethanol consumption, reflecting an initial event in the pathophysiology of ALD, possibly by controlling function of gut barrier.^43^ Although the mechanism by which alcohol consumption decreases the abundance of *A. muciniphila* is currently unclear, it was suggested that recovery of alcohol‐induced depletion of *A. muciniphila* by oral intake signifies a new therapeutic approach for ALD patients.^43^ A study showed that oral intake of *A. muciniphila* significantly alleviate hepatic injury induced by alcohol.^43^ In our study, in addition to striking increase of *A. muciniphila*, we also observed that berberine did not show protection in PGF mice, indicating that the pharmacological effect of berberine is gut‐flora‐dependent. Therefore, we might conclude that berberine might suppress the progression of ALD via regulation of flora communities, predominantly by enhancing the abundance of *A. muciniphila*. Moreover, altered composition of intestinal flora might be involved in facilitating G‐MDSC‐like cells expansion to attenuate alcoholic hepatic damage. Investigation on understanding how gut flora orchestrates the response of G‐MDSC‐like population is on‐going in our group.

## 5 CONCLUSIONS

This study revealed that berberine considerably prohibited acute‐on‐chronic alcoholic hepatic damage in mice via regulation of G‐MDSC‐like population and intestinal flora. We found that G‐MDSC‐like cells expanded remarkably after excessive alcohol exposure with declining amplitude along with long‐term drinking, whereas berberine maintained the increase and functioning of G‐MDSC‐like population partially by mediating IL‐6/STAT3 signaling pathways. Furthermore, it retrieved ethanol‐caused *A. muciniphila* reduction to facilitate the expansion of this immunosuppressive cell population, consequently prevented ALD. Collectively, our study positively proposed berberine that targets the gut microbiota‐immune system axis as a promising agent for ALD therapy.

## AUTHOR CONTRIBUTIONS

Sha Li performed the experiments, analyzed the data, and drafted the manuscript; Ning Wang, Hor‐Yue Tan, and Fan Chueng analyzed the data and revised the manuscript; Zhang‐jin Zhang and Man‐Fung Yuen revised the manuscript; and Yibin Feng designed the experiments, analyzed the data, and revised the manuscript. All authors approved the final version of the article, including the authorship list.

## CONFLICT OF INTEREST

The authors have declared no conflict of interest.

## Supporting information

SUPPORTING INFORMATION

SUPPORTING INFORMATION

SUPPORTING INFORMATION

SUPPORTING INFORMATION

SUPPORTING INFORMATION

## Data Availability

The data that support the findings of this study are available from the corresponding author upon reasonable request.

## References

[1] Louvet A , Mathurin P . Alcoholic liver disease: mechanisms of injury and targeted treatment. Nat Rev Gastroenterol Hepatol. 2015;12(4):231‐242. 25782093 10.1038/nrgastro.2015.35

[2] Gao B , Bataller R . Alcoholic liver disease: pathogenesis and new therapeutic targets. Gastroenterology. 2011;141(5):1572‐1585. 21920463 10.1053/j.gastro.2011.09.002 PMC3214974

[3] Sutti S , Bruzzi S , Albano E . The role of immune mechanisms in alcoholic and nonalcoholic steatohepatitis: a 2015 update. Expert Rev Gastroenterol Hepatol. 2016;10(2):243‐253. 26634783 10.1586/17474124.2016.1111758

[4] Albano E , Vidali M . Immune mechanisms in alcoholic liver disease. Genes Nutr. 2010;5(2):141‐147. 19809845 10.1007/s12263-009-0151-4 PMC2885168

[5] Gabrilovich DI , Nagaraj S . Myeloid‐derived suppressor cells as regulators of the immune system. Nat Rev Immunol. 2009;9(3):162‐174. 19197294 10.1038/nri2506 PMC2828349

[6] Ugel S , Delpozzo F , Desantis G , et al. Therapeutic targeting of myeloid‐derived suppressor cells. Curr Opin Pharmacol. 2009;9(4):470‐481. 19616475 10.1016/j.coph.2009.06.014

[7] Talmadge JE , Gabrilovich DI . History of myeloid‐derived suppressor cells. Nat Rev Cancer. 2013;13(10):739‐752. 24060865 10.1038/nrc3581 PMC4358792

[8] Bronte V . Myeloid‐derived suppressor cells in inflammation: uncovering cell subsets with enhanced immunosuppressive functions. Eur J Immunol. 2009;39(10):2670‐2672. 19757440 10.1002/eji.200939892

[9] Diao W , Jin F , Wang B , et al. The protective role of myeloid‐derived suppressor cells in concanavalin A‐induced hepatic injury. Protein Cell. 2014;5(9):714‐724. 24981055 10.1007/s13238-014-0069-5 PMC4145084

[10] Hegde VL , Nagarkatti PS , Nagarkatti M . Role of myeloid‐derived suppressor cells in amelioration of experimental autoimmune hepatitis following activation of TRPV1 receptors by cannabidiol. PLoS One. 2011;6(4):e18281. 21483776 10.1371/journal.pone.0018281 PMC3069975

[11] Guan Q , Blankstein AR , Anjos K , et al. Functional myeloid‐derived suppressor cell subsets recover rapidly after allogeneic hematopoietic stem/progenitor cell transplantation. Biol Blood Marrow Transplant. 2015;21(7):1205‐1214. 25963921 10.1016/j.bbmt.2015.04.015

[12] Dumitru CA , Moses K , Trellakis S , Lang S , Brandau S . Neutrophils and granulocytic myeloid‐derived suppressor cells: immunophenotyping, cell biology and clinical relevance in human oncology. Cancer Immunol Immunother. 2012;61(8):1155‐1167. 22692756 10.1007/s00262-012-1294-5 PMC11028504

[13] Bronte V , Brandau S , Chen S‐H , et al. Recommendations for myeloid‐derived suppressor cell nomenclature and characterization standards. Nat Commun. 2016;7:12150. 27381735 10.1038/ncomms12150 PMC4935811

[14] Pillay J , Tak T , Kamp VM , Koenderman L . Immune suppression by neutrophils and granulocytic myeloid‐derived suppressor cells: similarities and differences. Cell Mol Life Sci. 2013;70(20):3813‐3827. 23423530 10.1007/s00018-013-1286-4 PMC3781313

[15] Li S , Wang N , Tan HY , et al. Expansion of granulocytic, myeloid‐derived suppressor cells in response to ethanol‐induced acute liver damage. Front Immunol. 2018;9:1524. 30072984 10.3389/fimmu.2018.01524 PMC6060237

[16] Tang J , Feng Y , Tsao S , Wang N , Curtain R , Wang Y . Berberine and Coptidis rhizoma as novel antineoplastic agents: a review of traditional use and biomedical investigations. J Ethnopharmacol. 2009;126(1):5‐17. 19686830 10.1016/j.jep.2009.08.009

[17] Wang N , Feng Y , Zhu M , et al. Berberine induces autophagic cell death and mitochondrial apoptosis in liver cancer cells: the cellular mechanism. J Cell Biochem. 2010;111(6):1426‐1436. 20830746 10.1002/jcb.22869

[18] Wang N , Zhu M , Wang X , Tan HY , Tsao SW , Feng Y . Berberine‐induced tumor suppressor p53 up‐regulation gets involved in the regulatory network of MIR‐23a in hepatocellular carcinoma. Biochim Biophys Acta. 2014;1839(9):849‐857. 24942805 10.1016/j.bbagrm.2014.05.027

[19] Wang N , Xu Q , Tan HY , et al. Berberine inhibition of fibrogenesis in a rat model of liver fibrosis and in hepatic stellate cells. Evid Based Complement Alternat Med. 2016;2016:8762345. 27239214 10.1155/2016/8762345 PMC4867075

[20] Zhang Z , Li B , Meng X , et al. Berberine prevents progression from hepatic steatosis to steatohepatitis and fibrosis by reducing endoplasmic reticulum stress. Sci Rep. 2016;6:20848. 26857750 10.1038/srep20848 PMC4746620

[21] Zhang P , Ma D , Wang Y , et al. Berberine protects liver from ethanol‐induced oxidative stress and steatosis in mice. Food Chem Toxicol. 2014;74:225‐232. 25455889 10.1016/j.fct.2014.10.005

[22] Bertola A , Mathews S , Ki SW , Wang H , Gao B . Mouse model of chronic and binge ethanol feeding (the NIAAA model). Nat Protoc. 2013;8(3):627‐637. 23449255 10.1038/nprot.2013.032 PMC3788579

[23] Szabo G . Gut‐liver axis in alcoholic liver disease. Gastroenterology. 2015;148(1):30‐36. 25447847 10.1053/j.gastro.2014.10.042 PMC4274189

[24] Szabo G , Petrasek J . Gut‐liver axis and sterile signals in the development of alcoholic liver disease. Alcohol Alcohol. 2017;52(4):414‐424. 28482064 10.1093/alcalc/agx025 PMC5860369

[25] Szabo G , Bala S . Alcoholic liver disease and the gut‐liver axis. World J Gastroenterol. 2010;16(11):1321‐1329. 20238398 10.3748/wjg.v16.i11.1321 PMC2842523

[26] Scarpellini E , Forlino M , Lupo M , et al. Gut microbiota and alcoholic liver disease. Rev Recent Clin Trials. 2016;11(3):213‐219. 27515958 10.2174/1574887111666160810100538

[27] Wang Y , Shou HW , Li XY , et al. Berberine‐induced bioactive metabolites of the gut microbiota improve energy metabolism. Metabolism. 2017;70:72‐84. 28403947 10.1016/j.metabol.2017.02.003

[28] Feng R , Shou JW , Zhao ZX , et al. Transforming berberine into its intestine‐absorbable form by the gut microbiota. Sci Rep. 2015;5:12155. 26174047 10.1038/srep12155 PMC4502414

[29] Wang K , Feng X , Chai L , Cao S , Qiu F . The metabolism of berberine and its contribution to the pharmacological effects. Drug Metab Rev. 2017;49(2):139‐157. 28290706 10.1080/03602532.2017.1306544

[30] Sander LE , Sackett SD , Dierssen U , et al. Hepatic acute‐phase proteins control innate immune responses during infection by promoting myeloid‐derived suppressor cell function. J Exp Med. 2010;207(7):1453‐1464. 20530204 10.1084/jem.20091474 PMC2901069

[31] Xia S , Sha H , Yang L , Ji Y , Ostrand‐Rosenberg S , Qi L . Gr‐1+ CD11b+ myeloid‐derived suppressor cells suppress inflammation and promote insulin sensitivity in obesity. J Biol Chem. 2011;286(26):23591‐23599. 21592961 10.1074/jbc.M111.237123 PMC3123122

[32] Dubinkina VB , Tyakht AV , Odintsova VY , et al. Links of gut microbiota composition with alcohol dependence syndrome and alcoholic liver disease. Microbiome. 2017;5(1):141. 29041989 10.1186/s40168-017-0359-2 PMC5645934

[33] Li S , Wang N , Hong M , Tan HY , Pan G , Feng Y . Hepatoprotective effects of a functional formula of three chinese medicinal herbs: experimental evidence and network pharmacology‐based identification of mechanism of action and potential bioactive components. Molecules. 2018;23(2):352. 10.3390/molecules23020352 PMC6017312 29414910

[34] Wang N , Tan HY , Li S , Feng Y . Atg9b deficiency suppresses autophagy and potentiates endoplasmic reticulum stress‐associated hepatocyte apoptosis in hepatocarcinogenesis. Theranostics. 2017;7(8):2325‐2338. 28740555 10.7150/thno.18225 PMC5505064

[35] Kawaratani H , Tsujimoto T , Douhara A , et al. The effect of inflammatory cytokines in alcoholic liver disease. Mediators Inflamm. 2013;2013:495156. 24385684 10.1155/2013/495156 PMC3872233

[36] Mandrekar P , Ambade A . Immunity and inflammatory signaling in alcoholic liver disease. Hepatol Int. 2014;8(Suppl 2):439‐446. 26201323 10.1007/s12072-014-9518-8 PMC4587491

[37] Garg A , Spector SA . HIV type 1 gp120‐induced expansion of myeloid derived suppressor cells is dependent on interleukin 6 and suppresses immunity. J Infect Dis. 2014;209(3):441‐451. 23999600 10.1093/infdis/jit469 PMC3883171

[38] Zhang X , Zhao Y , Zhang M , et al. Structural changes of gut microbiota during berberine‐mediated prevention of obesity and insulin resistance in high‐fat diet‐fed rats. PLoS One. 2012;7(8):e42529. 22880019 10.1371/journal.pone.0042529 PMC3411811

[39] Zhu L , Zhang D , Zhu H , et al. Berberine treatment increases Akkermansia in the gut and improves high‐fat diet‐induced atherosclerosis in Apoe(‐/‐) mice. Atherosclerosis. 2018;268:117‐126. 29202334 10.1016/j.atherosclerosis.2017.11.023

[40] Cao Y , Pan Q , Cai W , et al. Modulation of gut microbiota by berberine improves steatohepatitis in high‐fat diet‐fed BALB/C mice. Arch Iran Med. 2016;19(3):197‐203. 26923892

[41] Fujio‐Vejar S , Vasquez Y , Morales P , et al. The gut microbiota of healthy chilean subjects reveals a high abundance of the phylum Verrucomicrobia. Front Microbiol. 2017;8:1221. 28713349 10.3389/fmicb.2017.01221 PMC5491548

[42] Shin NR , Whon TW , Bae JW . Proteobacteria: microbial signature of dysbiosis in gut microbiota. Trends Biotechnol. 2015;33(9):496‐503. 26210164 10.1016/j.tibtech.2015.06.011

[43] Grander C , Adolph TE , Wieser V , et al. Recovery of ethanol‐induced Akkermansia muciniphila depletion ameliorates alcoholic liver disease. Gut. 2017;67:891‐901. 28550049 10.1136/gutjnl-2016-313432

[44] Parker KH , Beury DW , Ostrand‐Rosenberg S . Myeloid‐derived suppressor cells: critical cells driving immune suppression in the tumor microenvironment. Adv Cancer Res. 2015;128:95‐139. 26216631 10.1016/bs.acr.2015.04.002 PMC4662416

[45] Guo C , Hu F , Yi H , et al. Myeloid‐derived suppressor cells have a proinflammatory role in the pathogenesis of autoimmune arthritis. Ann Rheum Dis. 2016;75(1):278‐285. 25371442 10.1136/annrheumdis-2014-205508 PMC4418961

[46] Dai J , El Gazzar M , Li GY , Moorman JP , Yao ZQ . Myeloid‐derived suppressor cells: paradoxical roles in infection and immunity. J Innate Immun. 2015;7(2):116‐126. 25401944 10.1159/000368233 PMC4348209

[47] Hammerich L , Tacke F . Emerging roles of myeloid derived suppressor cells in hepatic inflammation and fibrosis. World J Gastrointest Pathophysiol. 2015;6(3):43‐50. 26301117 10.4291/wjgp.v6.i3.43 PMC4540705

[48] Sumida K , Wakita D , Narita Y , et al. Anti‐IL‐6 receptor mAb eliminates myeloid‐derived suppressor cells and inhibits tumor growth by enhancing T‐cell responses. Eur J Immunol. 2012;42(8):2060‐2072. 22653638 10.1002/eji.201142335

[49] Chen MF , Kuan FC , Yen TC , et al. IL‐6‐stimulated CD11b+ CD14+ HLA‐DR‐ myeloid‐derived suppressor cells, are associated with progression and poor prognosis in squamous cell carcinoma of the esophagus. Oncotarget. 2014;5(18):8716‐8728. 25238263 10.18632/oncotarget.2368 PMC4226716

[50] Peng D , Tanikawa T , Li W , et al. Myeloid‐derived suppressor cells endow stem‐like qualities to breast cancer cells through IL6/STAT3 and NO/NOTCH cross‐talk signaling. Cancer Res. 2016;76(11):3156‐3165. 27197152 10.1158/0008-5472.CAN-15-2528 PMC4891237

[51] Yue SJ , Liu J , Wang WX , et al. Berberine treatment‐emergent mild diarrhea associated with gut microbiota dysbiosis. Biomed Pharmacother. 2019;116:109002. 31154270 10.1016/j.biopha.2019.109002

[52] Everard A , Belzer C , Geurts L , et al. Cross‐talk between Akkermansia muciniphila and intestinal epithelium controls diet‐induced obesity. Proc Natl Acad Sci U S A. 2013;110(22):9066‐9071. 23671105 10.1073/pnas.1219451110 PMC3670398

