# Supplementary figures and images for: Modulation of gut microbiota mediates berberine‐induced expansion of immuno‐suppressive cells to against alcoholic liver disease
Source: Clin Transl Med. 2020 Aug 13;10(4):e112. doi: 10.1002/ctm2.112 (PMC7438809; doi:10.1002/ctm2.112)

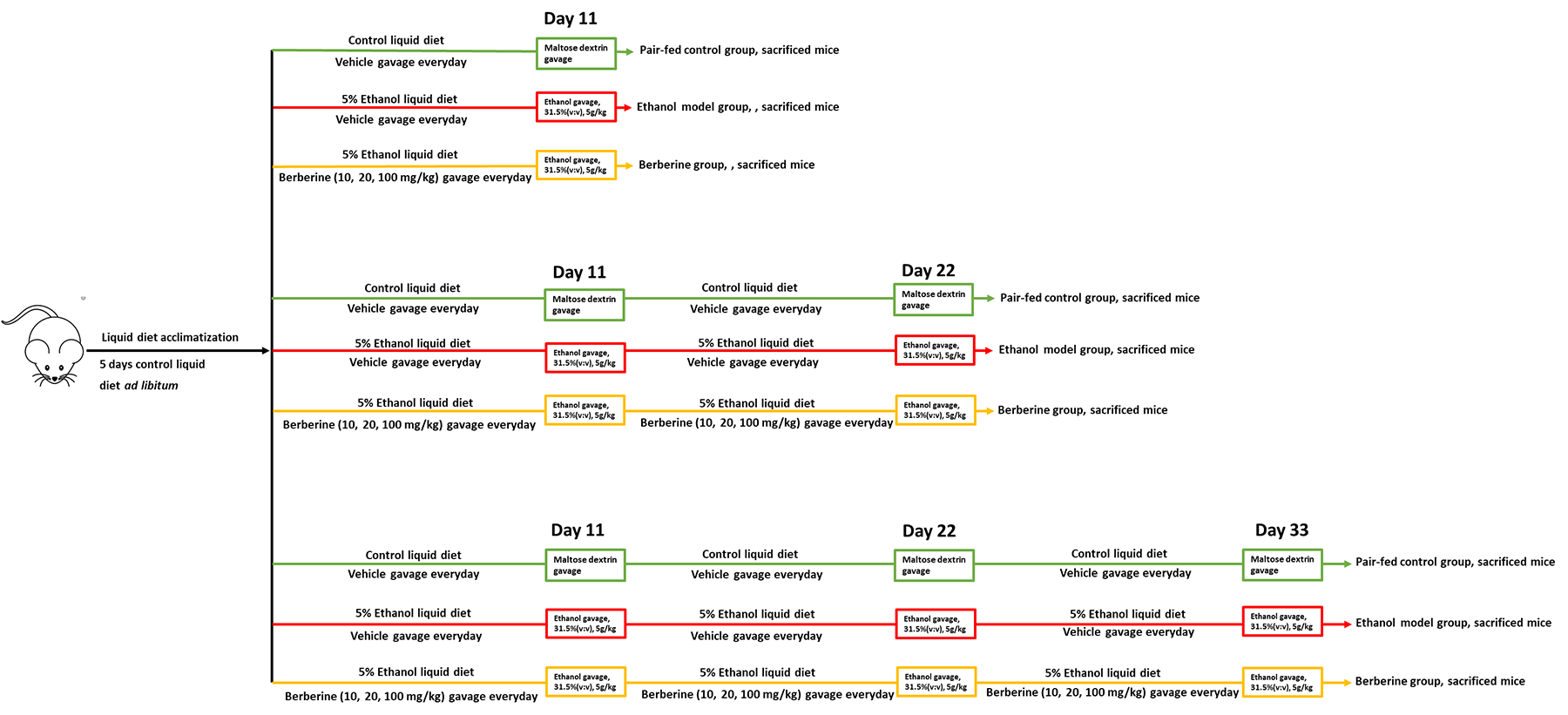

Supplement: Supplementary file 1 — SUPPORTING INFORMATION [file CTM2-10-e112-s001.tiff]

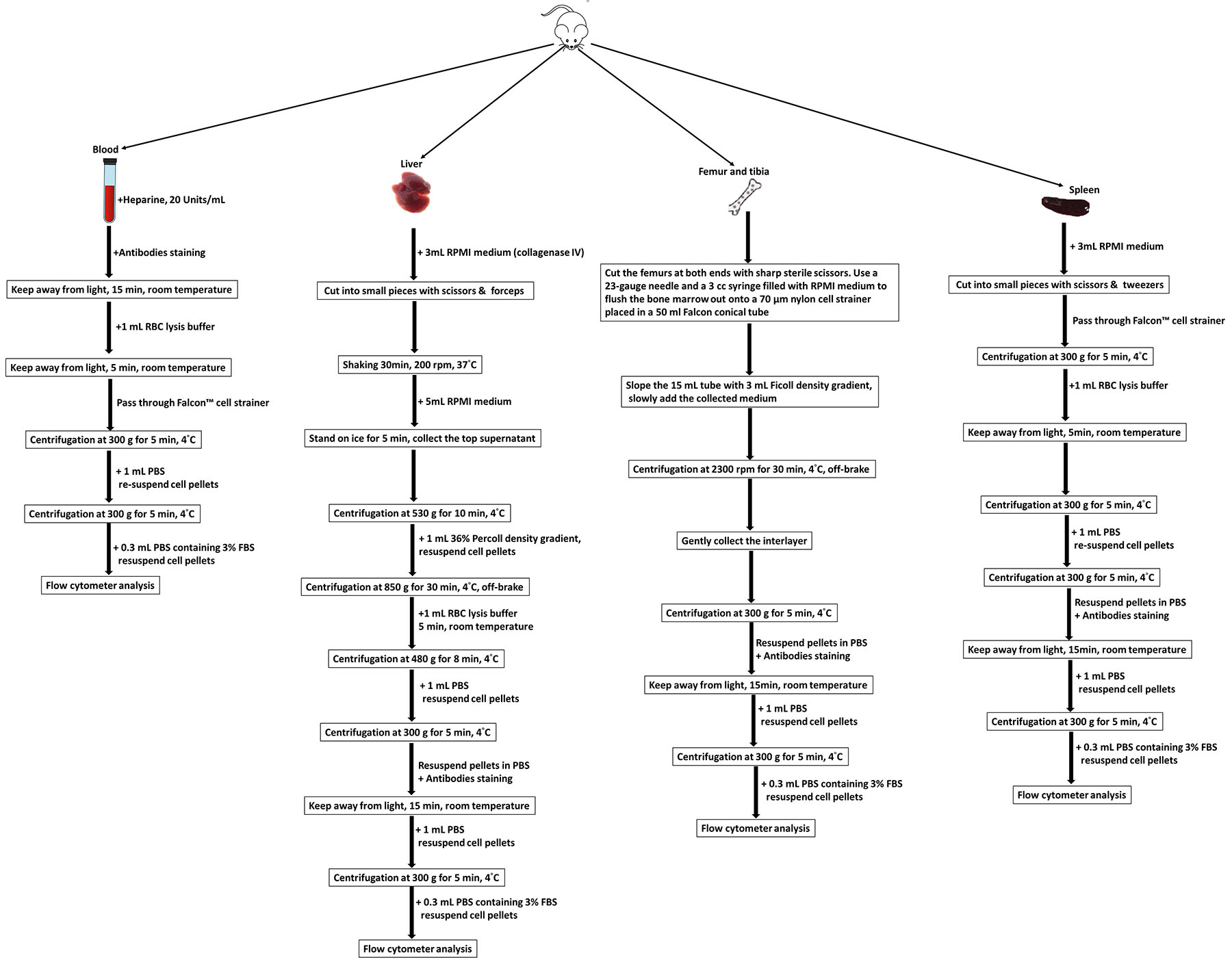

Supplement: Supplementary file 2 — SUPPORTING INFORMATION [file CTM2-10-e112-s002.jpg]

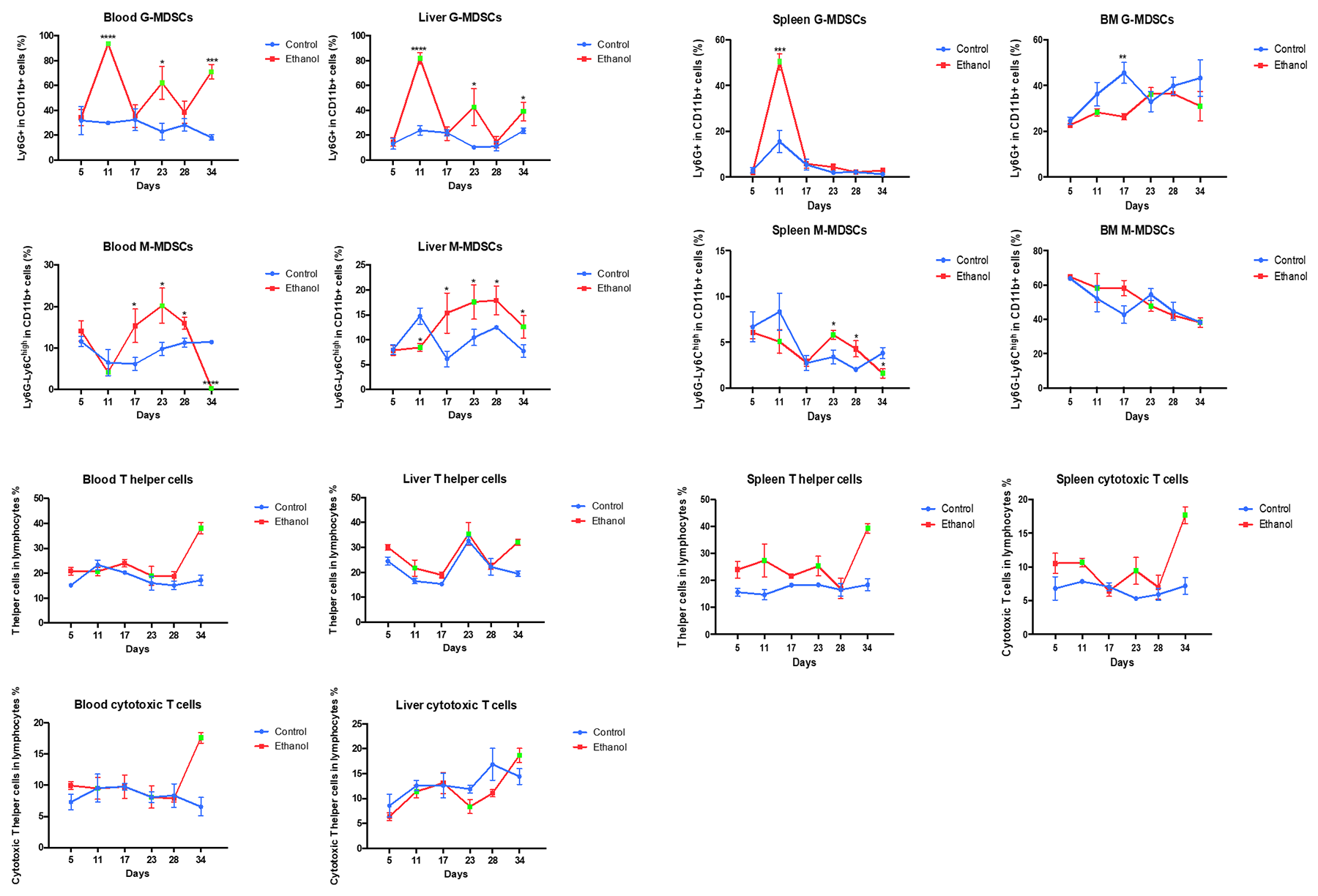

Supplement: Supplementary file 3 — SUPPORTING INFORMATION [file CTM2-10-e112-s003.tiff]

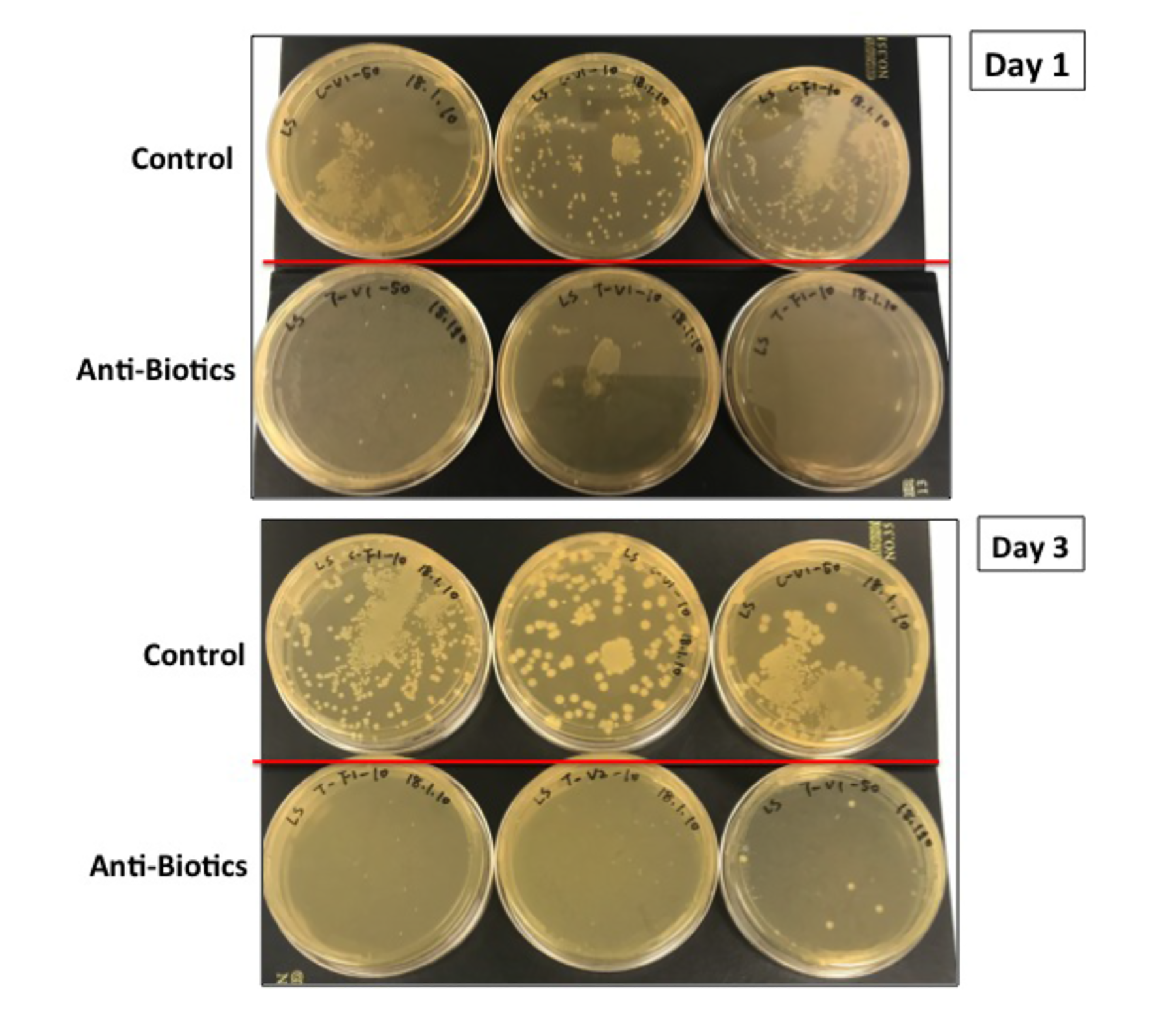

Supplement: Supplementary file 4 — SUPPORTING INFORMATION [file CTM2-10-e112-s004.tif]
