# Supplementary material for: Modulation of gut microbiota mediates berberine‐induced expansion of immuno‐suppressive cells to against alcoholic liver disease
Source: Clin Transl Med. 2020 Aug 13;10(4):e112. doi: 10.1002/ctm2.112 (PMC7438809; doi:10.1002/ctm2.112)
Supplement: Supplementary file 5 — SUPPORTING INFORMATION [file CTM2-10-e112-s005.docx]

| Name | Forward primer 5' -> 3' | Reverse primer 5' -> 3' |
| --- | --- | --- |
| β actin | GGCACCACACCTTCTACAATG | GGGGTGTTGAAGGTCTCAAAC |
| OCT4 | CCCCAATGCCGTGAAGTTG | TCAGCAGCTTGGCAAACTGTT |
| SOX-2 | CACAGATGCAACCGATGCA | GGTGCCCTGCTGCGAGTA |
| IL-10 | ATAACTGCACCCACTTCCCA | GGGCATCACTTCTACCAGGT |
| Arginase-1 | GGAATCTGCATGGGCAA | AGGGTCTACGTCTCGCAAGCCA |
| GM-CSF | TTTACTTTTCCTGGGCATTG | TAGCTGGCTGTCATGTTCAA |
| IL-6 | CCTCTGGTCTTCTGGAGTACC | ACTCCTTCTGTGACTCCAGC |
| sIL6R | GCGACAAGCCTCCCAGGTTC | GTGCCACCCAGCCAGCTATC |
| S100A8 | CCCGTCTTCAAGACATCGTTTG | ATATCCAGGGACCCAGCCCTAG |
| S100A9 | CGACACCTTCCATCAATACT | TCAGCATCATACTCCTCA |
| STAT3 | GGATCGCTGAGGTACAACCC | GTCAGGGGTCTCGACTGTCT |

**Supplemental Table 1** Quantitative PCR primers and corresponding sequences.
